# Supplementary material for: An Effective Model of the Retinoic Acid Induced HL-60 Differentiation Program
Source: Sci Rep. 2017 Oct 30;7:14327. doi: 10.1038/s41598-017-14523-5 (PMC5662654; doi:10.1038/s41598-017-14523-5)
Supplement: Supplementary file 1 — Supplemental Information [file 41598_2017_14523_MOESM1_ESM.pdf]

An Effective Model of the Retinoic Acid Induced HL-60 Differentiation Program

Ryan Tasseff, Holly A. Jensen, Johanna Congleton[‡], Wei Dai, Katharine V. Rogers, Adithya Sagar, Rodica P. Bunaciu[‡], Andrew Yen[‡] and Jeffrey D. Varner[*]

Robert Frederick Smith School of Chemical and Biomolecular Engineering

Cornell University, Ithaca NY 14853

[‡]Department of Biomedical Sciences

Cornell University, Ithaca NY 14853

[*]Corresponding author:

Jeffrey D. Varner,

Professor, Robert Frederick Smith School of Chemical and Biomolecular Engineering,

244 Olin Hall, Cornell University, Ithaca NY, 14853

Email: jdv27@cornell.edu

Supplemental materials.

Western and immunoprecipitation blots generated in this study.

List of Figures

S1 Western blot for phosphorylated ERK (total lysate) washout experiments. Blot corresponds to Fig 3C. S-4

S2 cRaf immunoprecipitation with c-Raf with and without ATRA treatment at T = 24 hr. Lanes one and two on replicate on the left correspond to Fig 8A, first row, first column. S-5

S3 Western blot for c-Raf (total lysate) with and without ATRA treatment at T = 24 hr. Blot corresponds to Fig 8A, first row, second column. S-6

S4 cRaf-PS621 immunoprecipitation with c-Raf with and without ATRA treatment at T = 24 hr. Blot corresponds to Fig 8A, second row, first column. . . S-7

S5 Western blot for c-Raf-PS621 (total lysate) with and without ATRA treatment at T = 24 hr. Blot corresponds to Fig 8A, second row, second column. S-8

S6 Immunoprecipitation study for the interaction of cRaf with Vav1 with and without ATRA treatment at T = 24 hr. Blot corresponds to Fig 8A, third row, first column. Also visible on Blot is a replicate for the total lysate measurement of Vav1 (right). S-9

S7 Western blot (total lysate) for Vav1 with and without ATRA treatment at T = 24 hr. Blot corresponds to Fig 8A, third row, second column. S-10

S8 Src immunoprecipitation with cRaf with and without ATRA treatment at T = 24 hr. Blot corresponds to Fig 8A, fourth row, first column. S-11

S9 Western blot Src (total lysate) with and without ATRA treatment at T = 24 hr (top, right-hand total panel, lanes: Lane 1 control, Lane 2 ATRA treatment). Blot corresponds to Fig 8A, fourth row, second column. S-12

S10 Akt immunoprecipitation with cRaf with and without ATRA treatment at T = 24 hr (top blot). Lanes one and two on the left correspond to Fig 8A, sixth row, first column. S-13
S11 Western blot (total lysate) for Akt with and without ATRA treatment at T = 24 hr. Blot corresponds to Fig 8A, sixth row, second column. S-14
S12 Western blot (total lysate) for b-Raf-pSer445 with and without ATRA treatment at T = 24 hr. Blot corresponds to Fig 8B, first row, first column. S-15
S13 Western blot (total lysate) for PKCα with and without ATRA treatment at T = 24 hr. Blot corresponds to Fig 8B, second row, first column. S-16
S14 Western blot (total lysate) for PKCγ with and without ATRA treatment at T = 24 hr. Blot corresponds to Fig 8B, third row, first column. S-17
S15 Western blot (total lysate) for PKA with and without ATRA treatment at T = 24 hr. Blot corresponds to Fig 8B, fourth row, first column. S-18
S16 Western blot (total lysate) for p38 with and without ATRA treatment at T = 24 hr. Blot corresponds to Fig 8B, fifth row, first column. S-19
S17 Western blot (total lysate) for Rac1/2/3 with and without ATRA treatment at T = 24 hr. Blot corresponds to Fig 8B, sixth row, first column. S-20
S18 Western blot (total lysate) for Cdc42 with and without ATRA treatment at T = 24 hr. Blot corresponds to Fig 8B, seventh row, first column. S-21
S19 Western blot (total lysate) for RhoB with and without ATRA treatment at T = 24 hr. Blot corresponds to Fig 8B, eight row, first column. S-22
S20 Western blot (total lysate) for Arrestin with and without ATRA treatment at T = 24 hr. Blot corresponds to Fig 8B, first row, second column. Lanes: In the total section, lane 1: control (no ATRA), lane 2: ATRA and lane 3: D3 treatment. S-23

S21 Western blot (total lysate) for Slp76 with and without ATRA treatment at T = 24 hr. Blot corresponds to Fig 8B, second row, second column. S-24
S22 Western blot (total lysate) for Cbl with and without ATRA treatment at T = 24 hr. Blot corresponds to Fig 8B, third row, second column. S-25
S23 Western blot (total lysate) for Rb with and without ATRA treatment at T = 24 hr. Blot corresponds to Fig 8B, fourth row, second column. S-26
S24 Western blot (total lysate) for PLCγ with and without ATRA treatment at T = 24 hr. Blot corresponds to Fig 8B, sixth row, second column. S-27
S25 Vav1 immunoprecipitation with cRaf with and without ATRA treatment and the Raf inhibitor GW5074 at T = 24 hr. Blot (right hand replicate) corresponds to Fig 8C, first row S-28
S26 Src immunoprecipitation with cRaf, and Src (total lysate) with and without ATRA treatment and the Raf inhibitor GW5074 at T = 24 hr (bottom replicate). Blot corresponds to Fig 8C, second row. Lanes: control (no ATRA or GW); ATRA alone; GW5074 alone; ATRA and GW5074. . . . . S-29
S27 14-3-3 immunoprecipitation with cRaf with and without ATRA treatment and the Raf inhibitor GW5074 at T = 24 hr. Blot corresponds to Fig 8C, third row S-30
S28 Akt immunoprecipitation with cRaf with and without ATRA treatment and the Raf inhibitor GW5074 at T = 24 hr. Blot corresponds to Fig 8C, fourth row S-31
S29 CK2 immunoprecipitation with cRaf with and without ATRA treatment and the Raf inhibitor GW5074 at T = 24 hr. Blot (right-hand replicate) corresponds to Fig 8C, fifth row S-32
S30 Western blot for P47Phox (total lysate) with and without ATRA treatment and the Raf inhibitor GW5074 at T = 48 hr. Blot (left-hand replicate) corresponds to Fig 8F. S-33

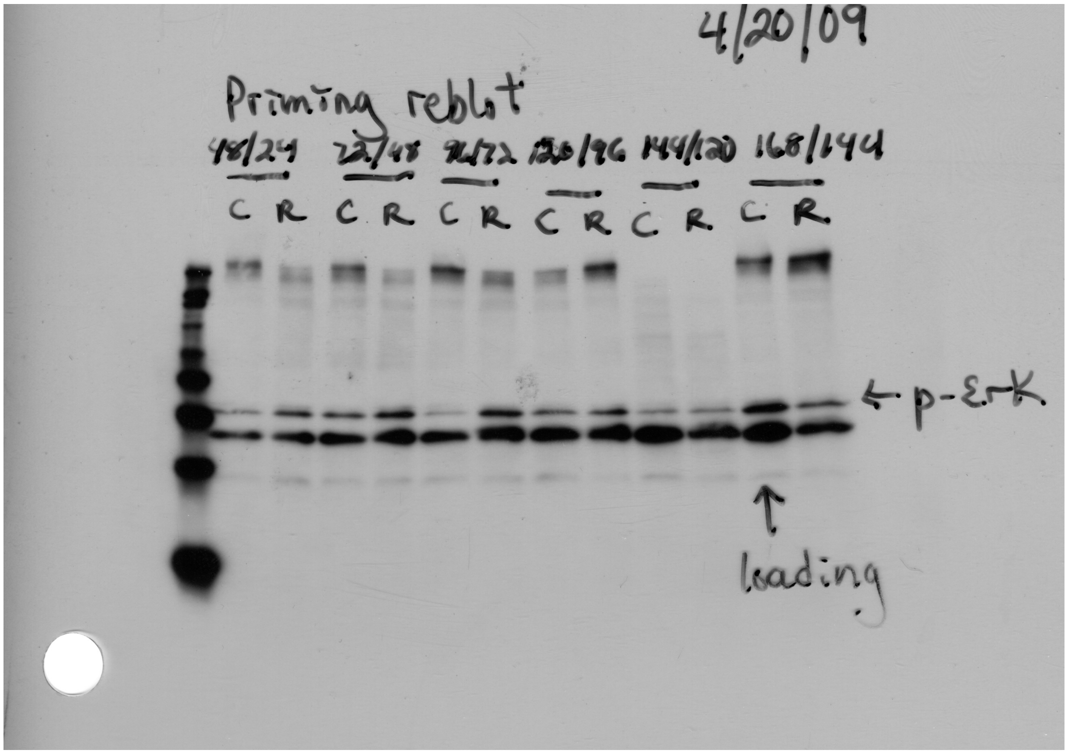

Fig. S1: Western blot for phosphorylated ERK (total lysate) washout experiments. Blot corresponds to Fig 3C.

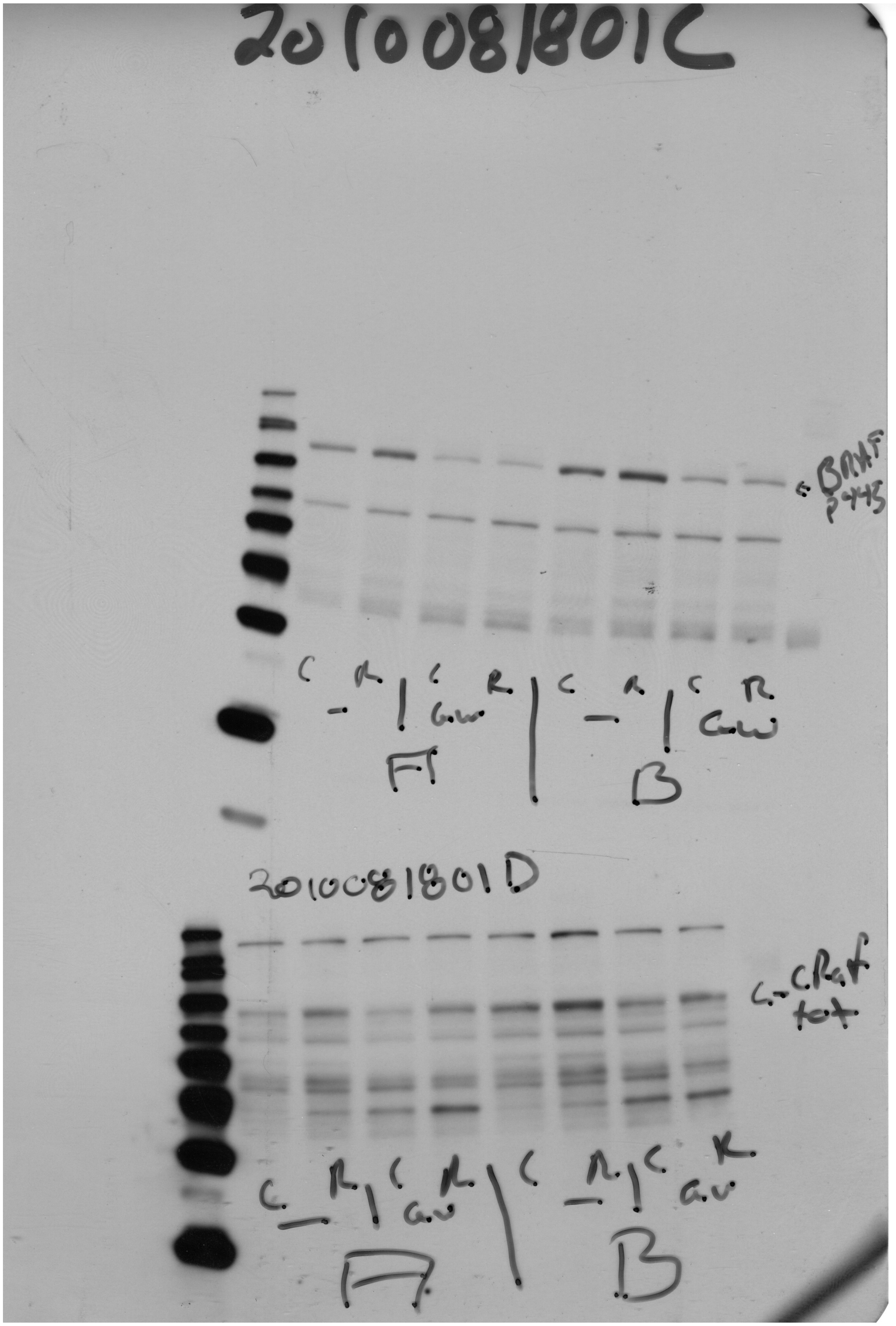

Fig. S2: cRaf immunoprecipitation with c-Raf with and without ATRA treatment at T = 24 hr. Lanes one and two on replicate on the left correspond to Fig 8A, first row, first column.

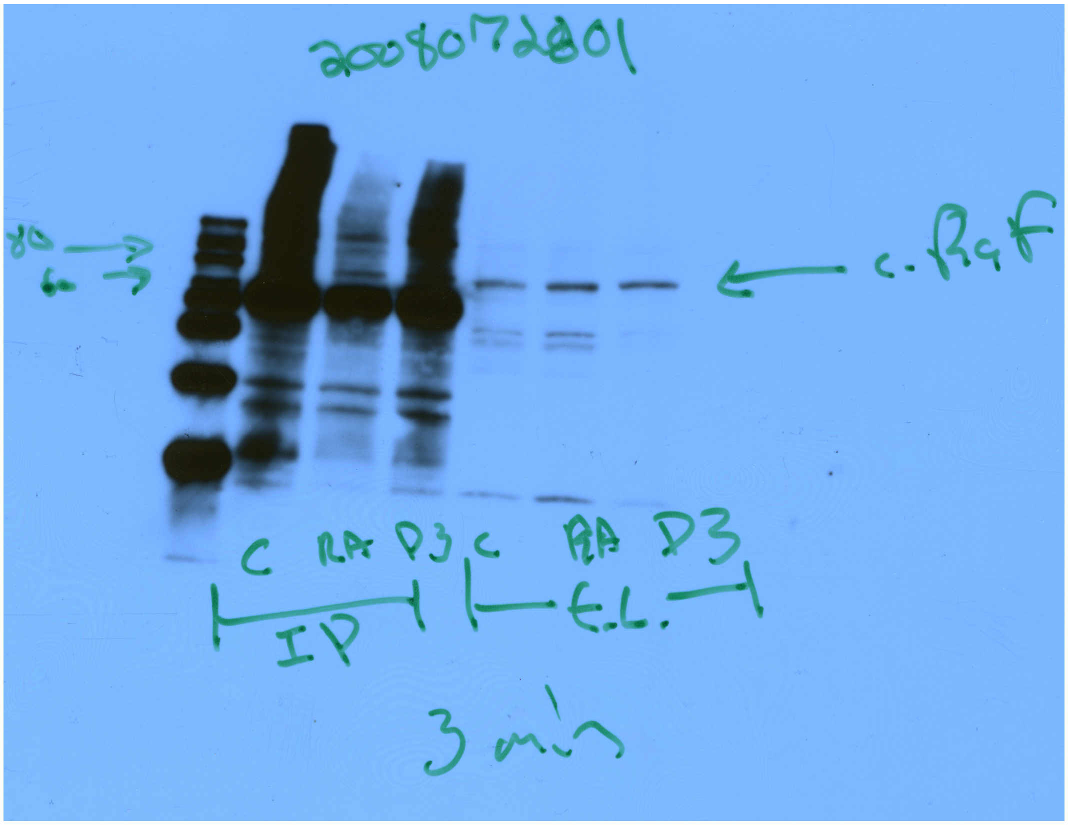

Fig. S3: Western blot for c-Raf (total lysate) with and without ATRA treatment at T = 24 hr. Blot corresponds to Fig 8A, first row, second column.

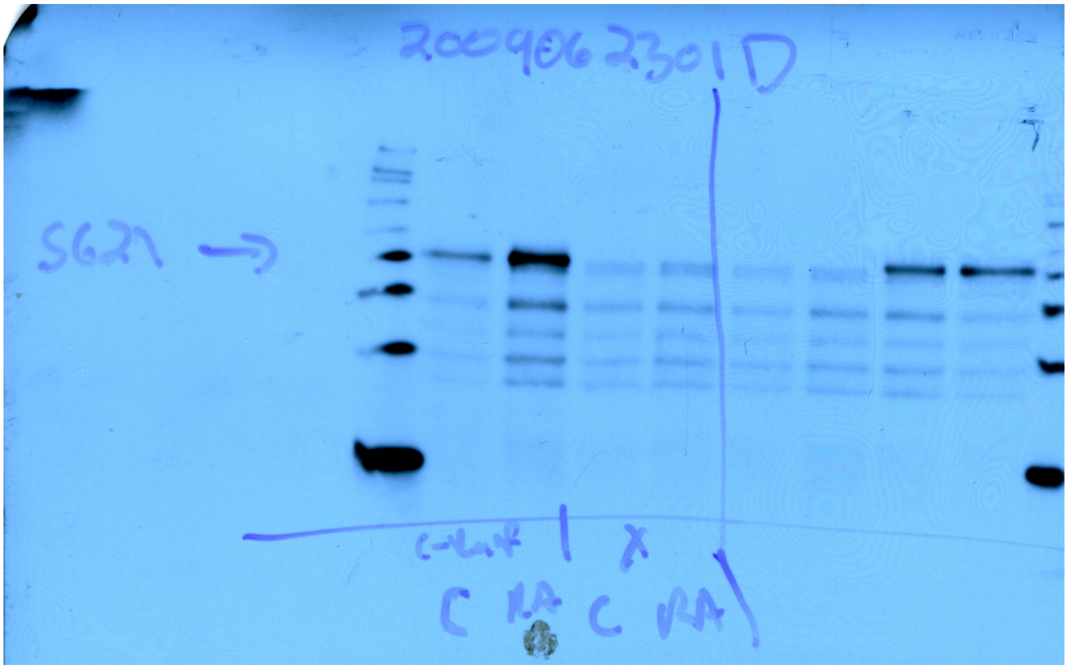

Fig. S4: cRaf-PS621 immunoprecipitation with c-Raf with and without ATRA treatment at T = 24 hr. Blot corresponds to Fig 8A, second row, first column.

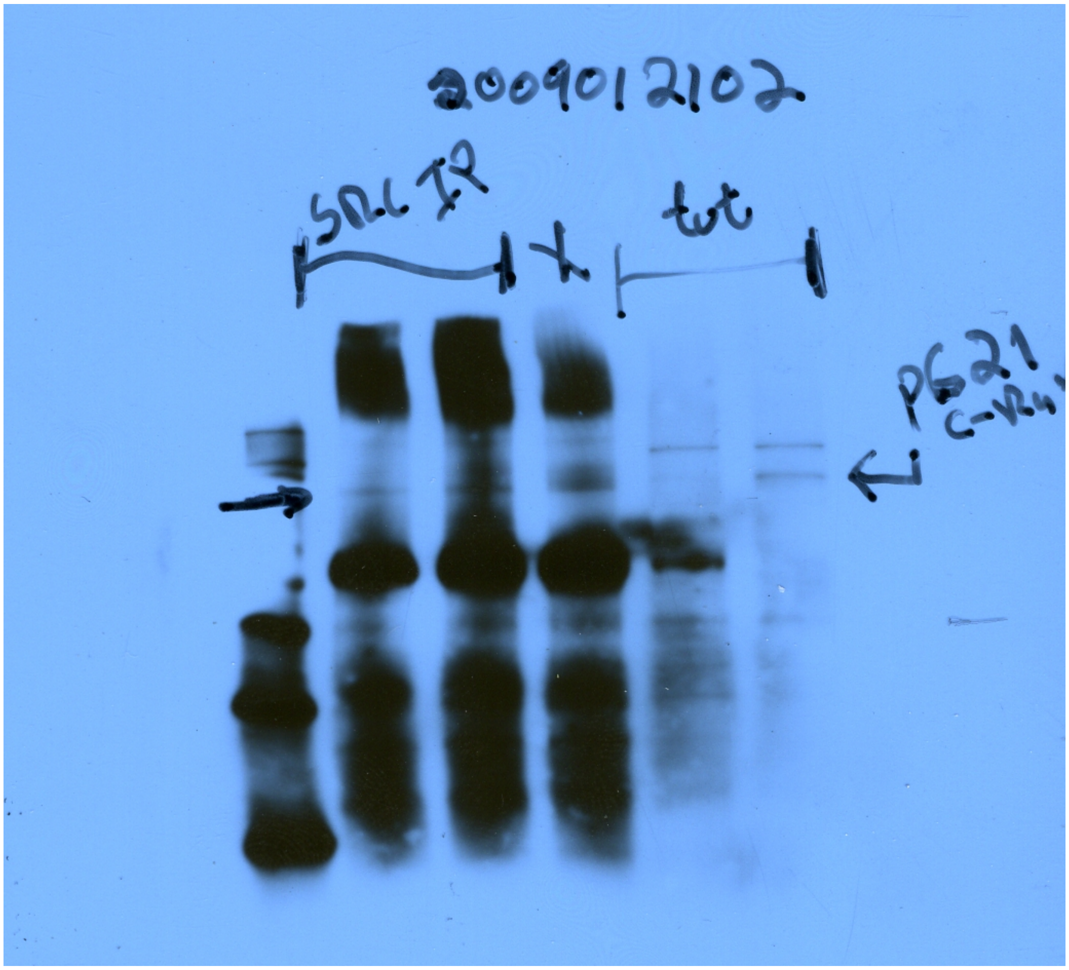

Fig. S5: Western blot for c-Raf-PS621 (total lysate) with and without ATRA treatment at T = 24 hr. Blot corresponds to Fig 8A, second row, second column.

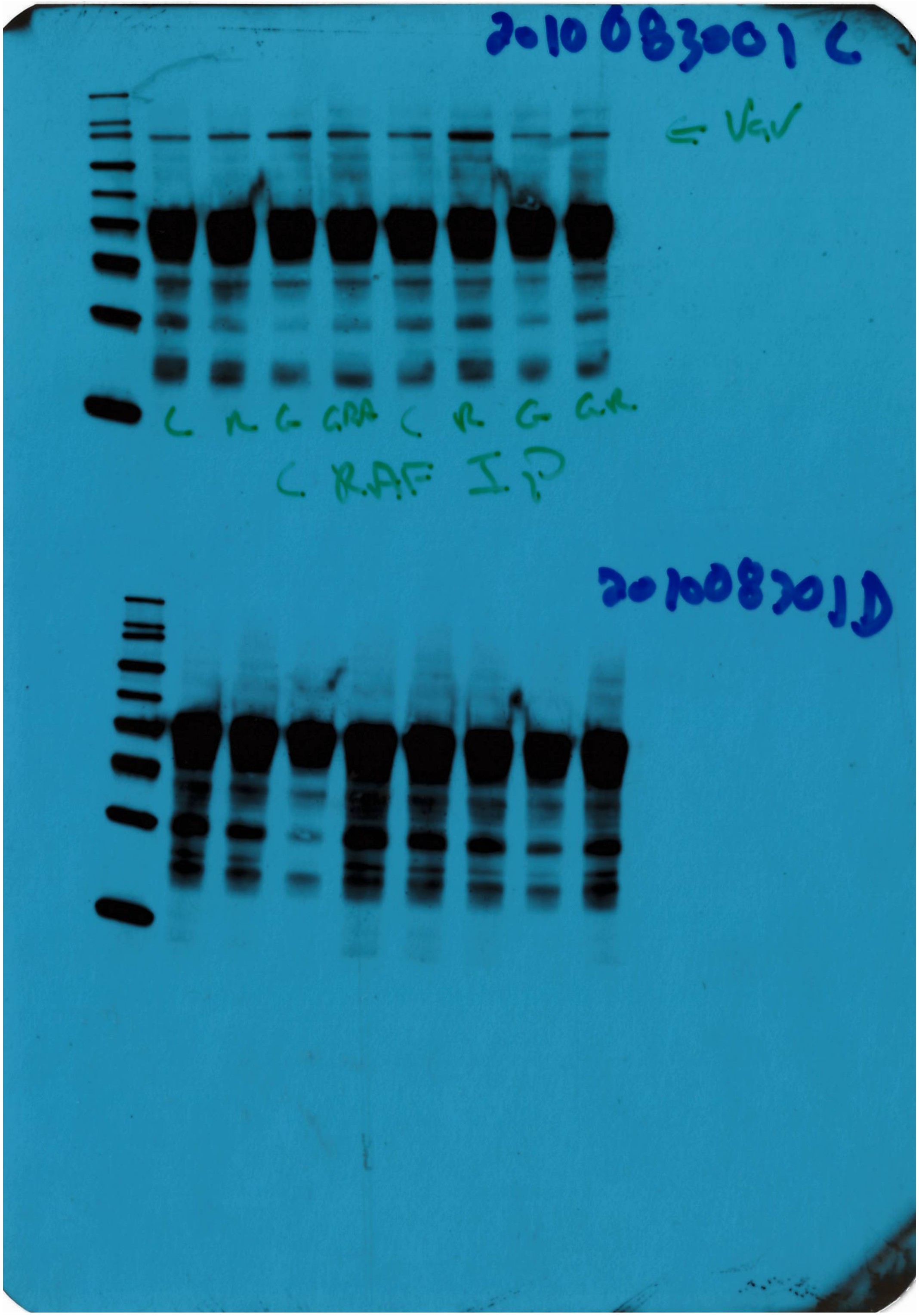

Fig. S6: Immunoprecipitation study for the interaction of cRaf with Vav1 with and without ATRA treatment at T = 24 hr. Blot corresponds to Fig 8A, third row, first column. Also visible on Blot is a replicate for the total lysate measurement of Vav1 (right).

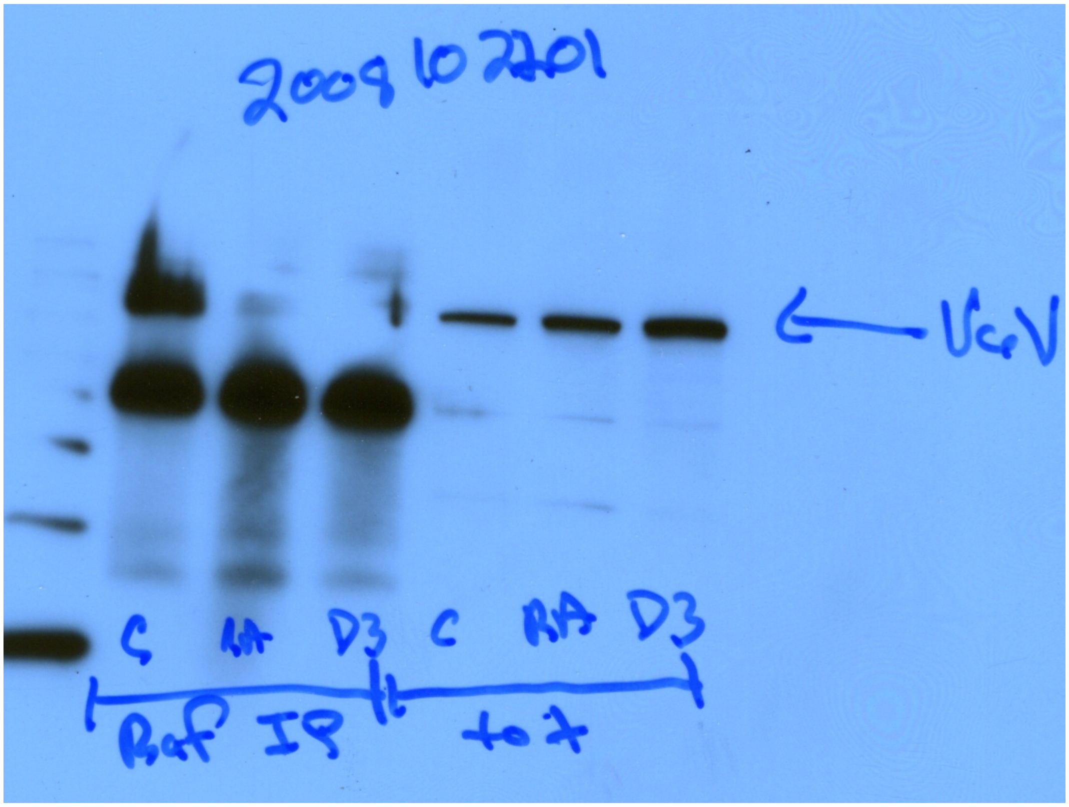

Fig. S7: Western blot (total lysate) for Vav1 with and without ATRA treatment at T = 24 hr. Blot corresponds to Fig 8A, third row, second column.

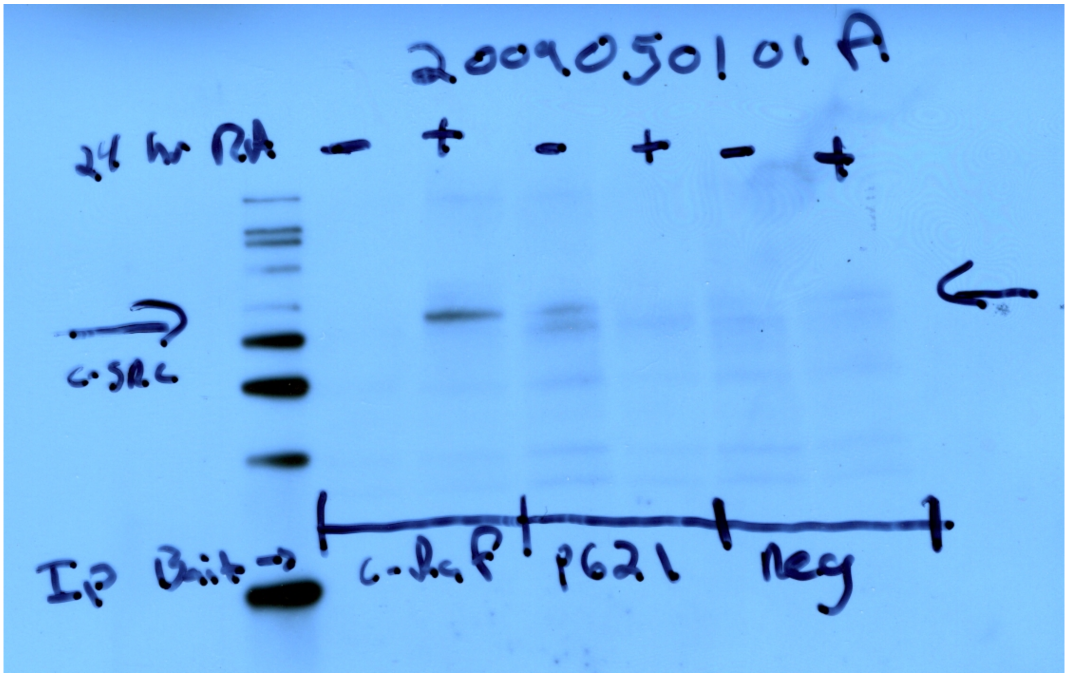

Fig. S8: Src immunoprecipitation with cRaf with and without ATRA treatment at T = 24 hr. Blot corresponds to Fig 8A, fourth row, first column.

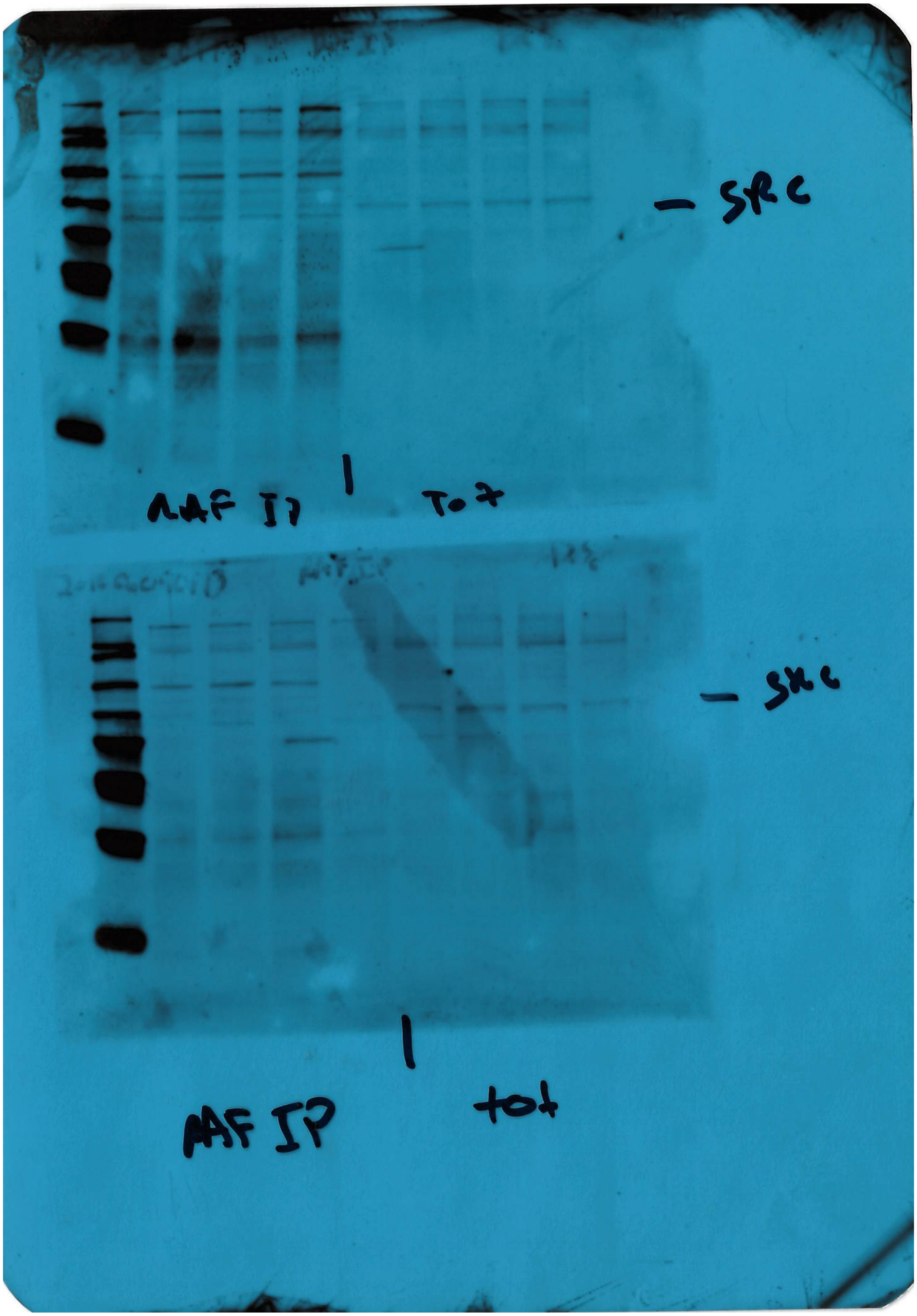

Fig. S9: Western blot Src (total lysate) with and without ATRA treatment at T = 24 hr (top, right-hand total panel, lanes: Lane 1 control, Lane 2 ATRA treatment). Blot corresponds to Fig 8A, fourth row, second column.

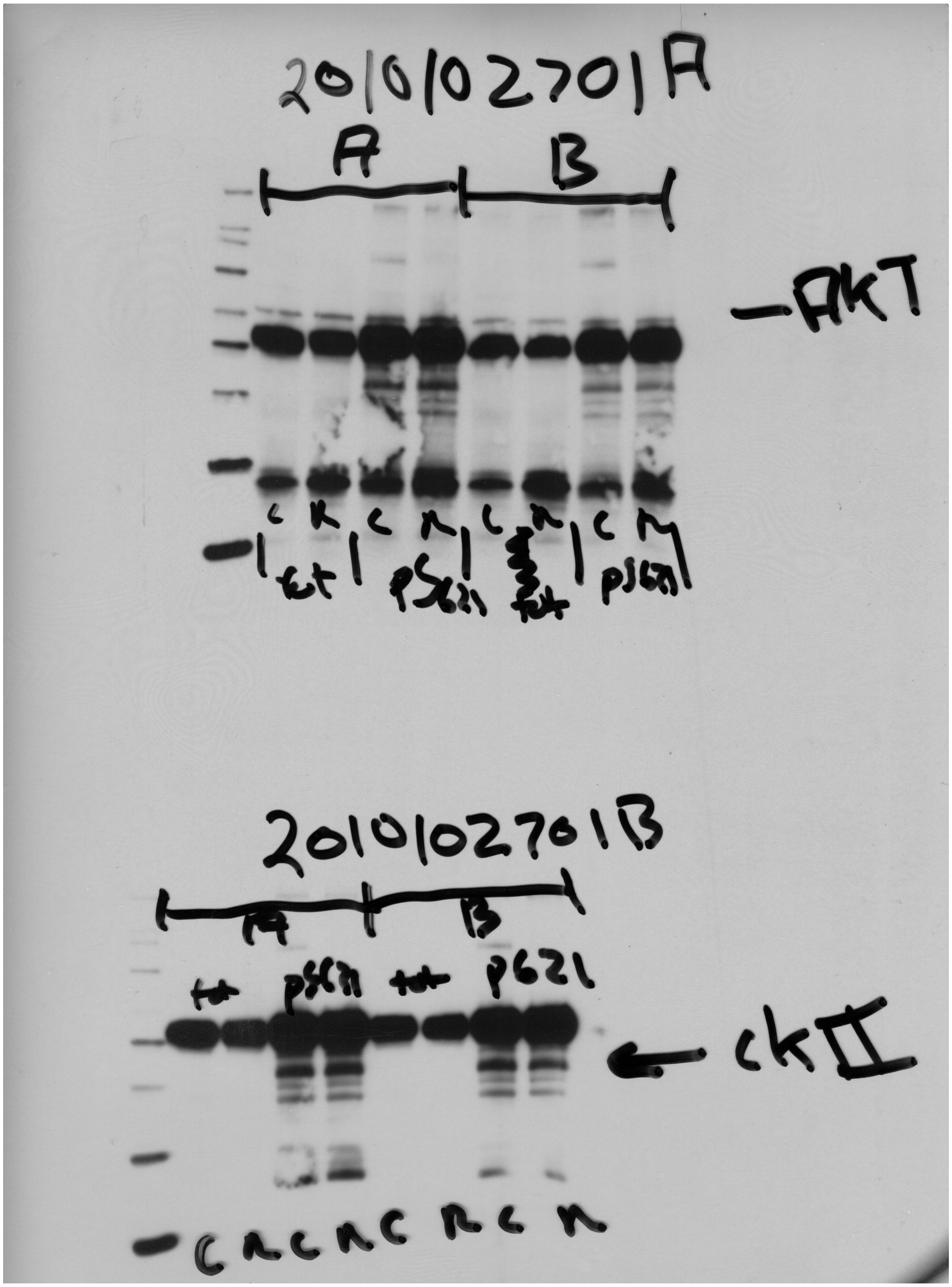

Fig. S10: Akt immunoprecipitation with cRaf with and without ATRA treatment at T = 24 hr (top blot). Lanes one and two on the left correspond to Fig 8A, sixth row, first column.

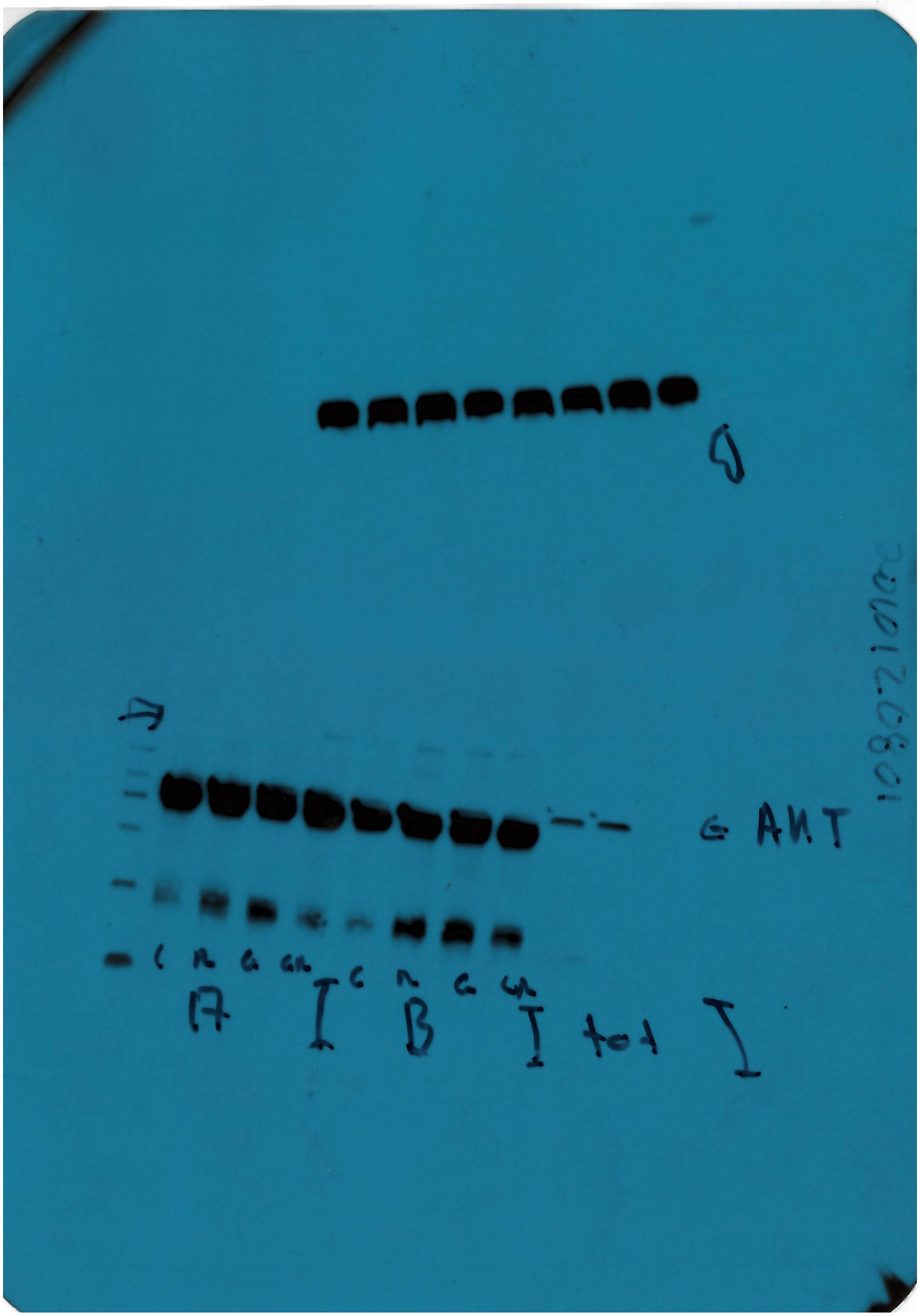

Fig. S11: Western blot (total lysate) for Akt with and without ATRA treatment at T = 24 hr. Blot corresponds to Fig 8A, sixth row, second column.

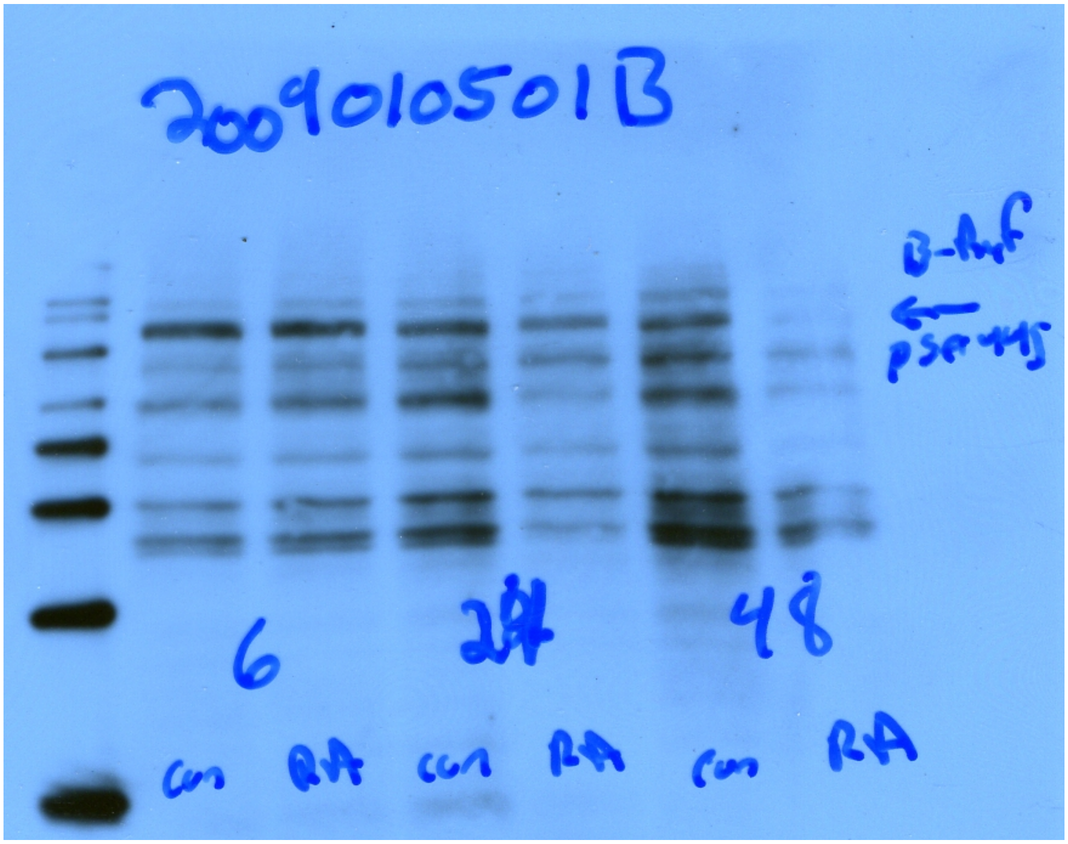

Fig. S12: Western blot (total lysate) for b-Raf-pSer445 with and without ATRA treatment at T = 24 hr. Blot corresponds to Fig 8B, first row, first column.

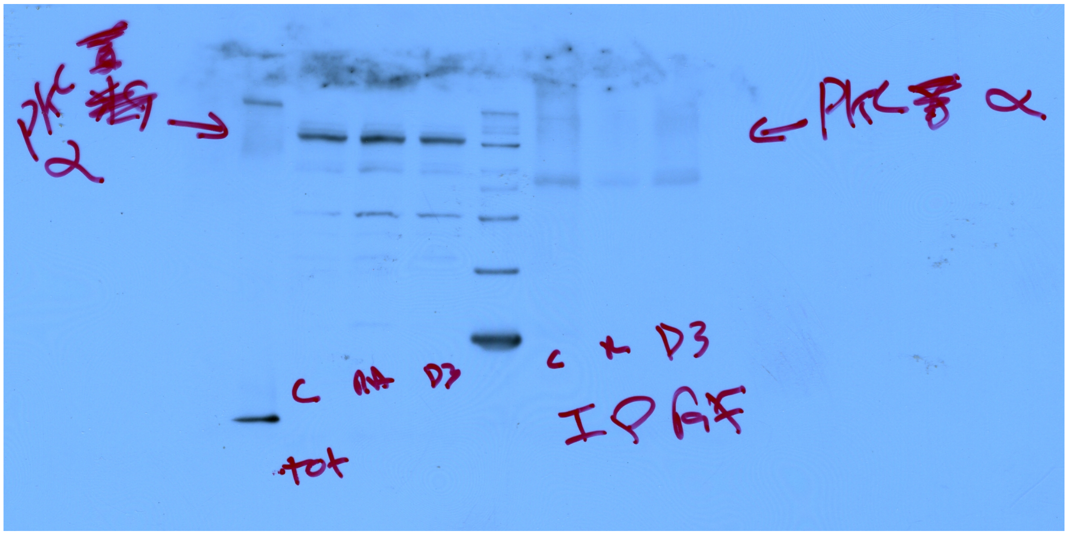

Fig. S13: Western blot (total lysate) for PKCα with and without ATRA treatment at T = 24 hr. Blot corresponds to Fig 8B, second row, first column.

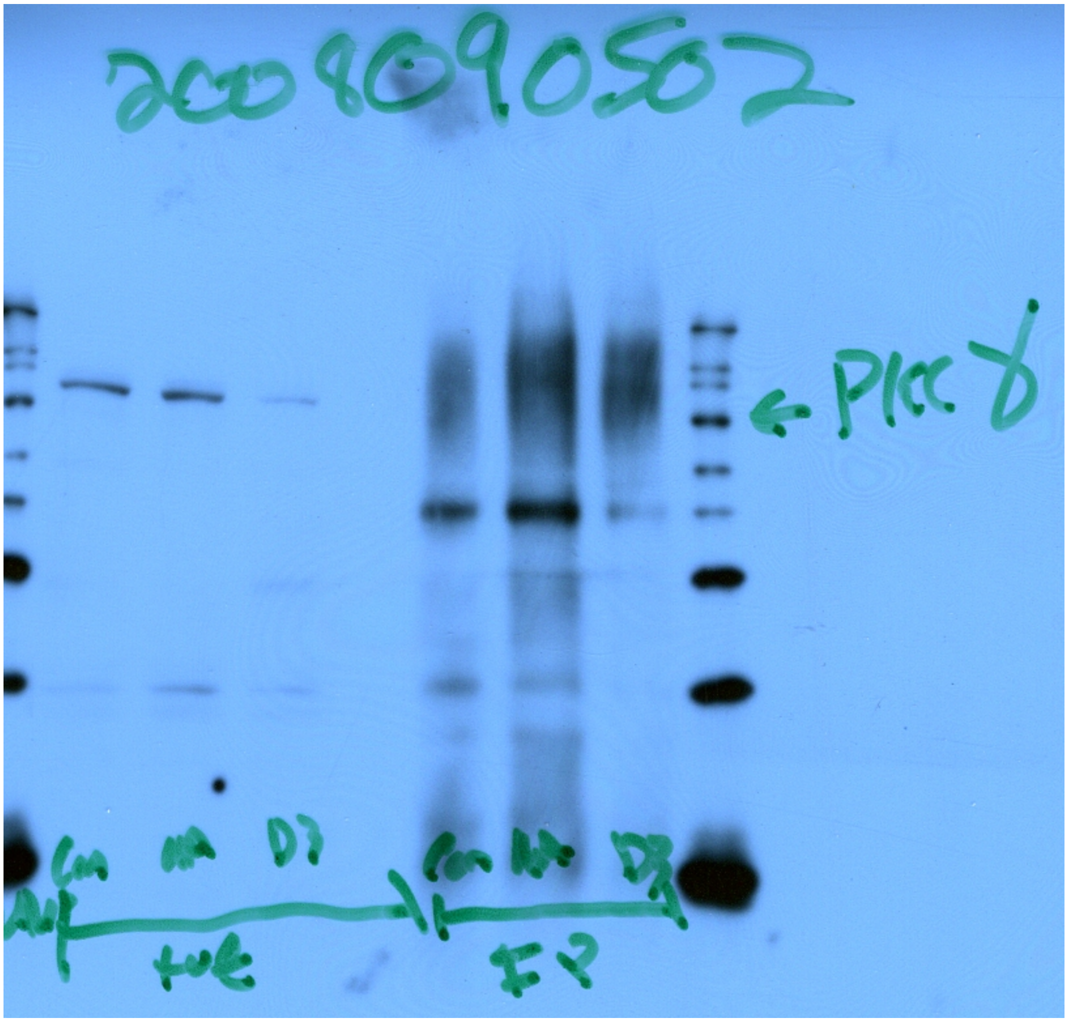

Fig. S14: Western blot (total lysate) for PKCγ with and without ATRA treatment at T = 24 hr. Blot corresponds to Fig 8B, third row, first column.

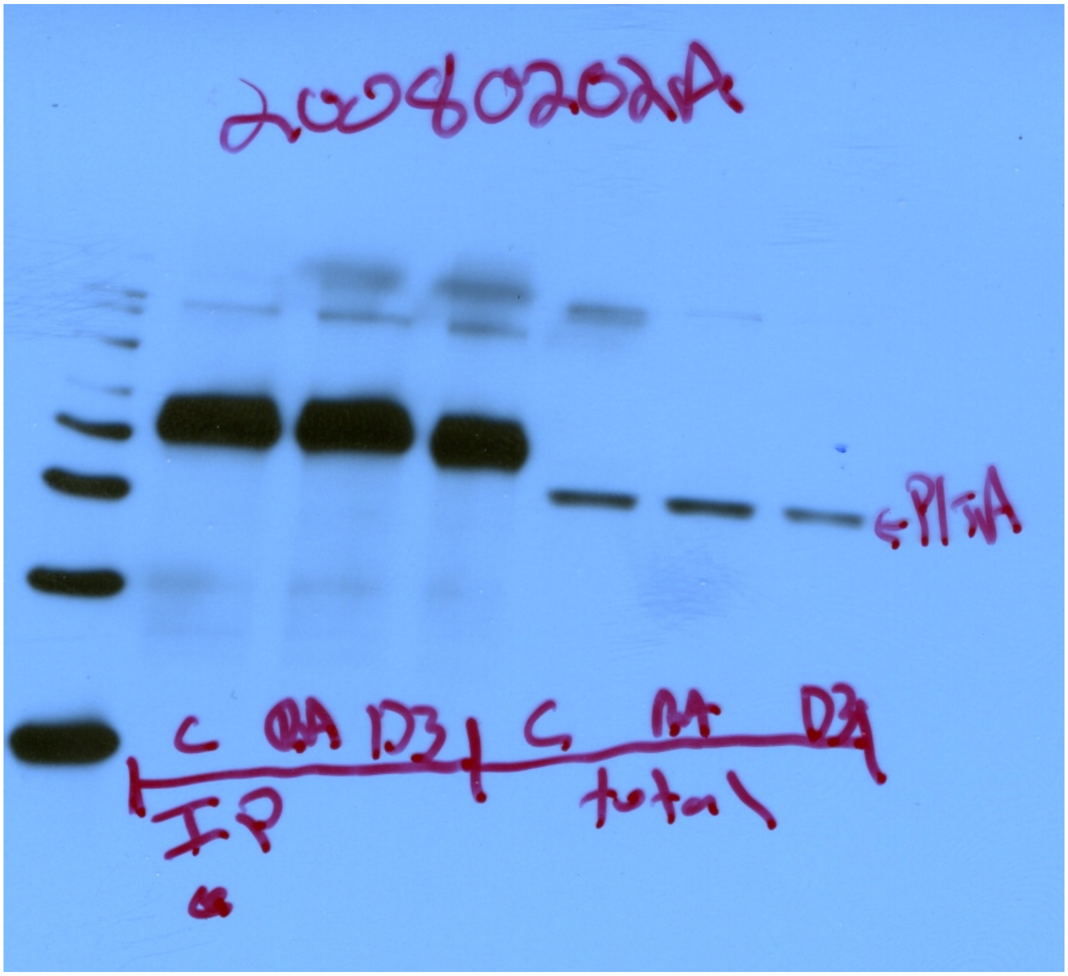

Fig. S15: Western blot (total lysate) for PKA with and without ATRA treatment at T = 24 hr. Blot corresponds to Fig 8B, fourth row, first column.

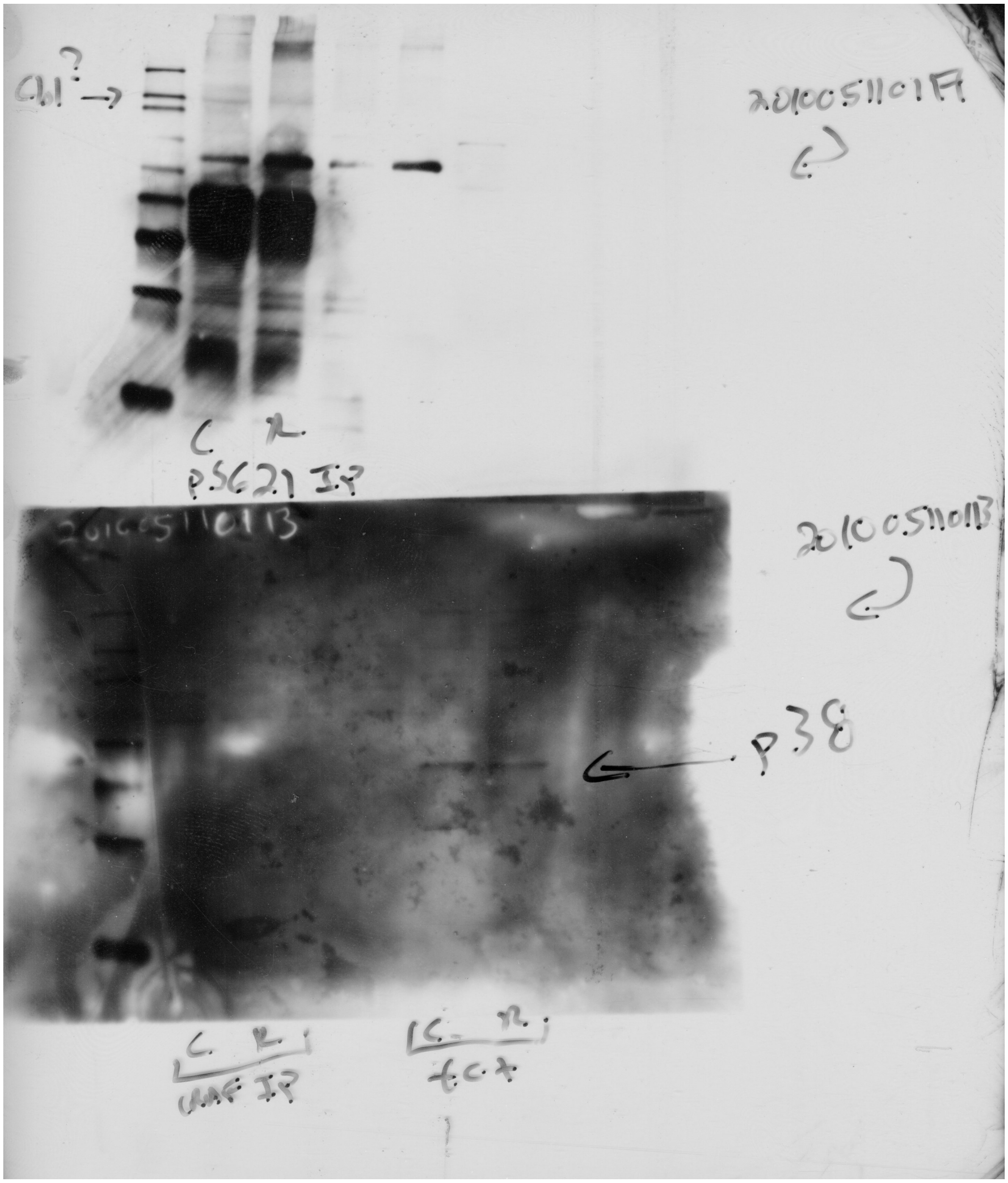

Fig. S16: Western blot (total lysate) for p38 with and without ATRA treatment at T = 24 hr. Blot corresponds to Fig 8B, fifth row, first column.

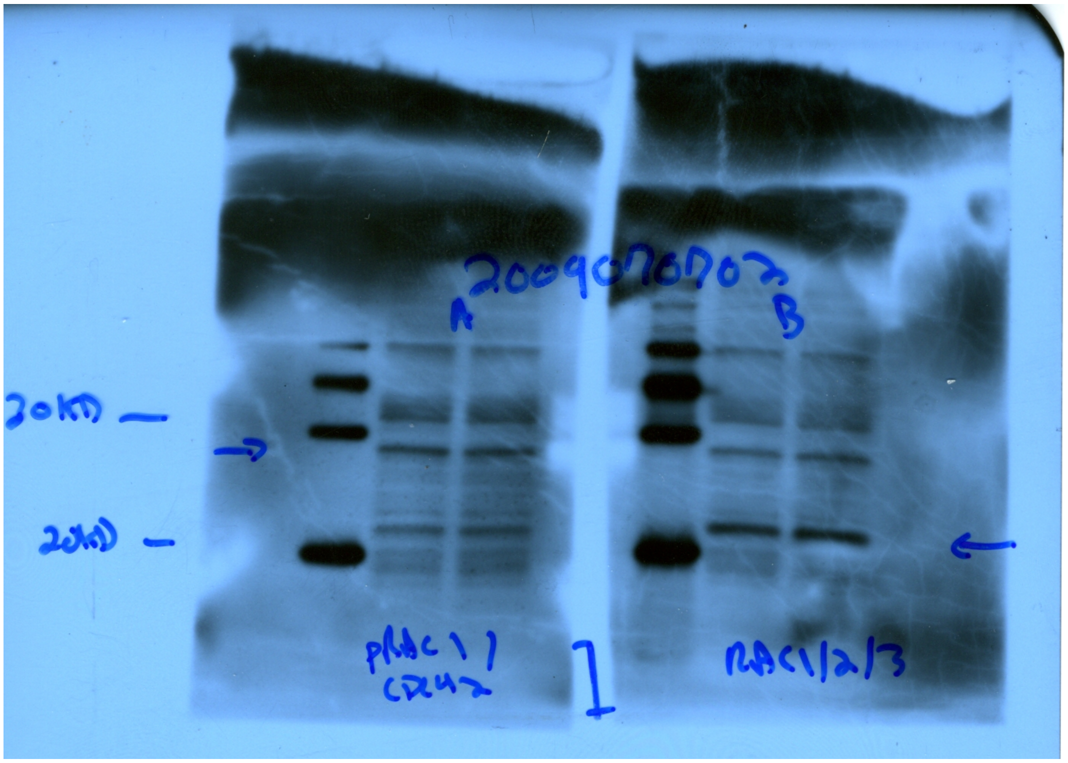

Fig. S17: Western blot (total lysate) for Rac1/2/3 with and without ATRA treatment at T = 24 hr. Blot corresponds to Fig 8B, sixth row, first column.

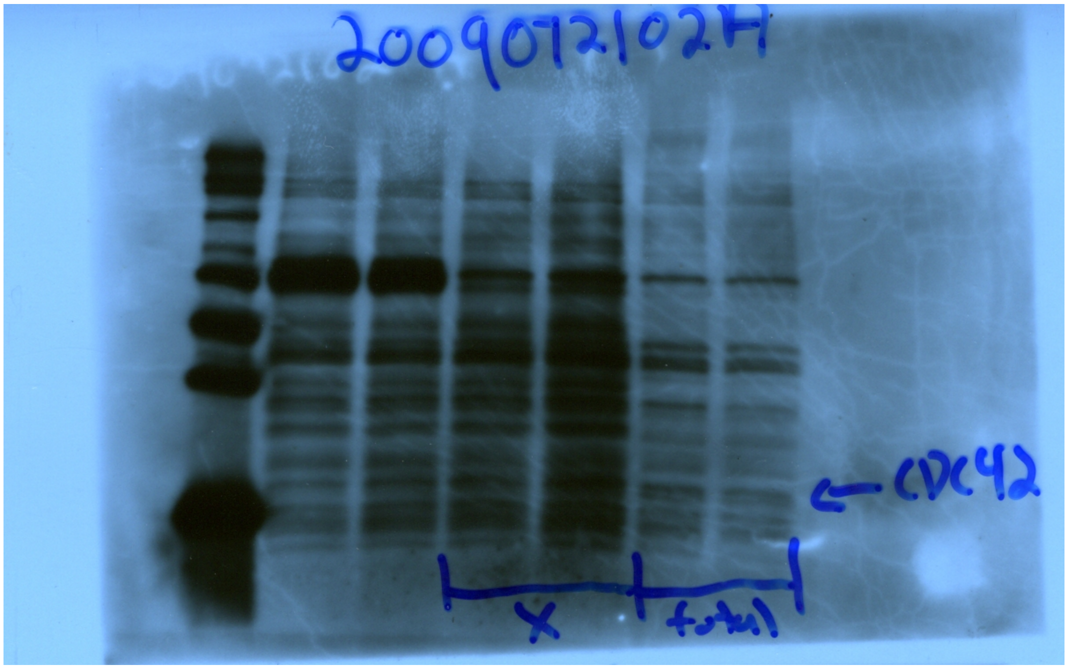

Fig. S18: Western blot (total lysate) for Cdc42 with and without ATRA treatment at T = 24 hr. Blot corresponds to Fig 8B, seventh row, first column.

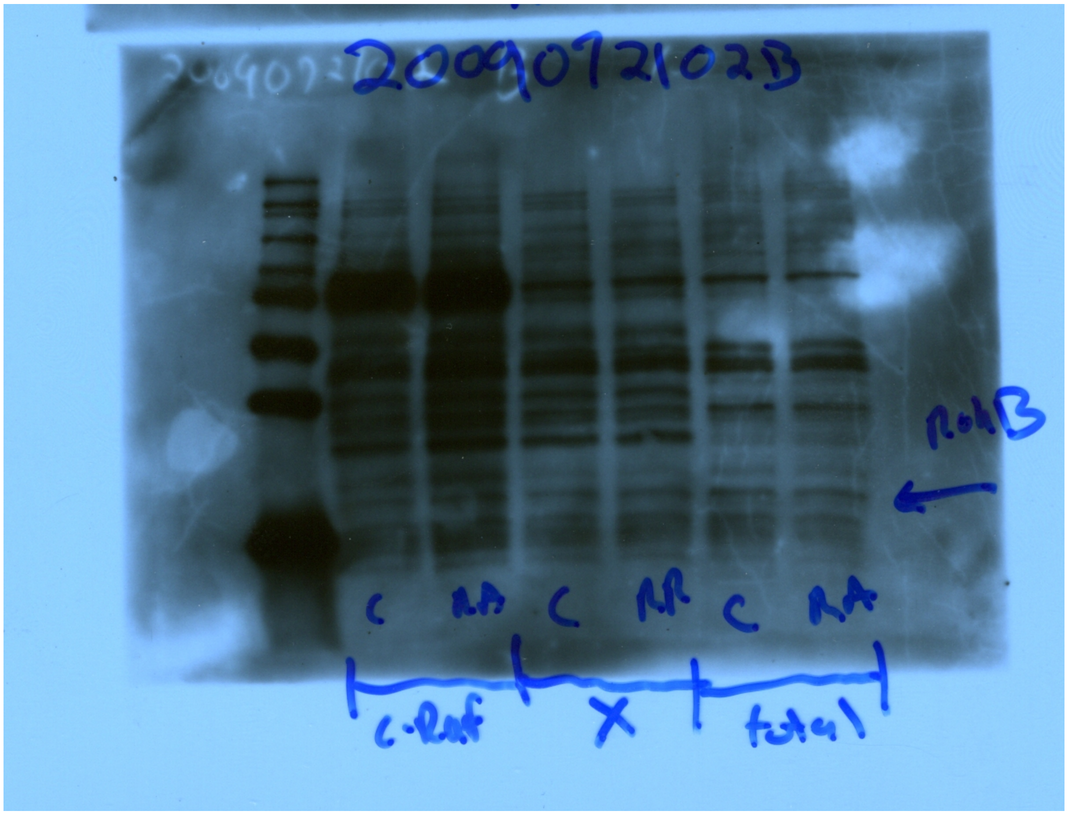

Fig. S19: Western blot (total lysate) for RhoB with and without ATRA treatment at T = 24 hr. Blot corresponds to Fig 8B, eight row, first column.

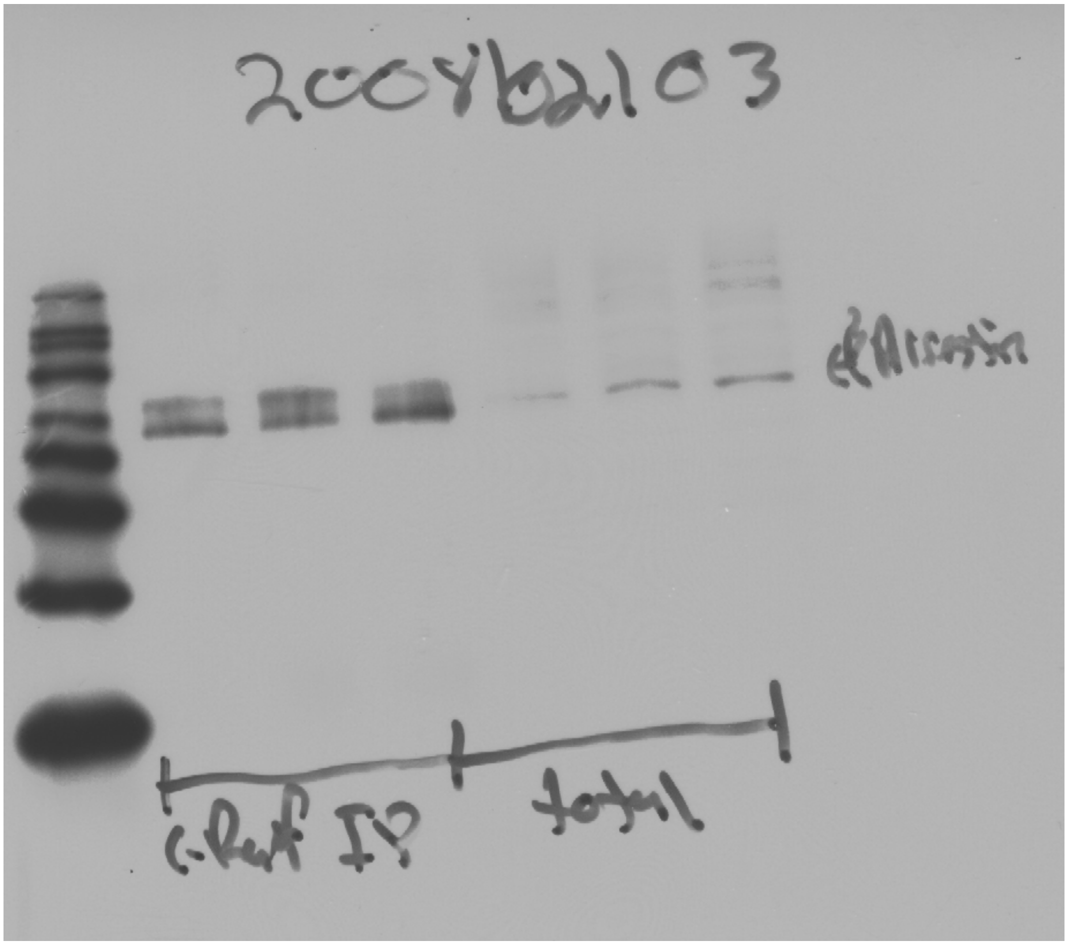

Fig. S20: Western blot (total lysate) for Arrestin with and without ATRA treatment at T = 24 hr. Blot corresponds to Fig 8B, first row, second column. Lanes: In the total section, lane 1: control (no ATRA), lane 2: ATRA and lane 3: D3 treatment.

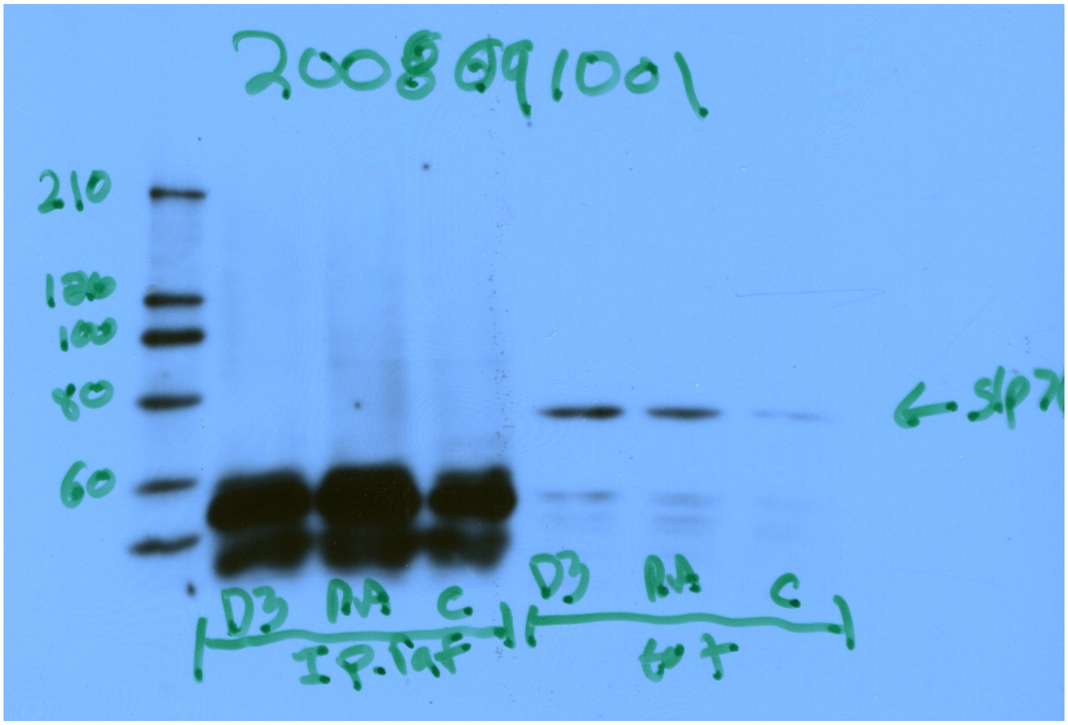

Fig. S21: Western blot (total lysate) for Slp76 with and without ATRA treatment at T = 24 hr. Blot corresponds to Fig 8B, second row, second column.

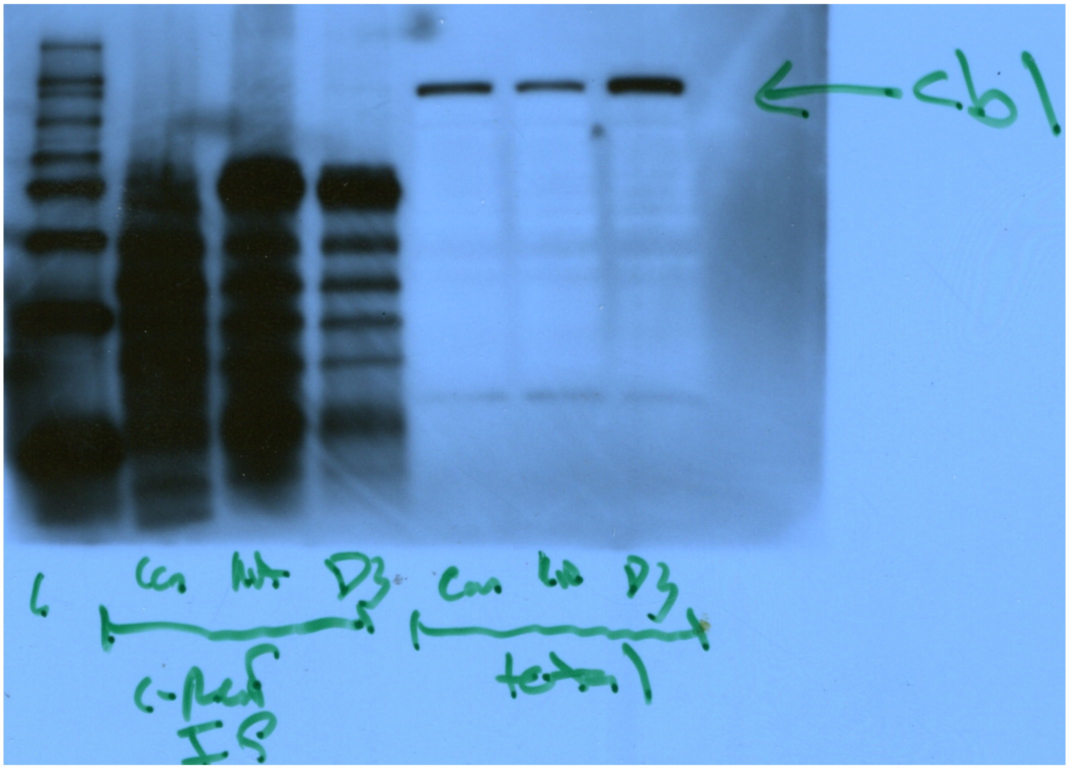

Fig. S22: Western blot (total lysate) for Cbl with and without ATRA treatment at T = 24 hr. Blot corresponds to Fig 8B, third row, second column.

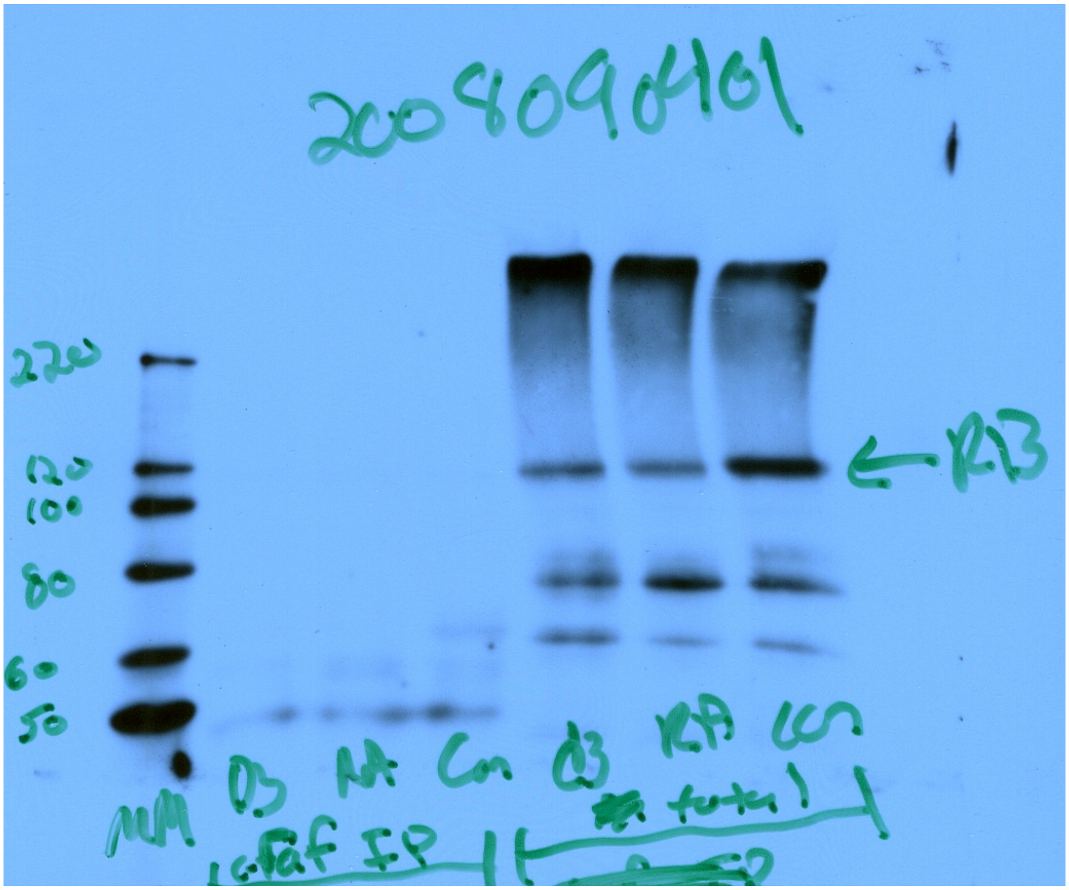

Fig. S23: Western blot (total lysate) for Rb with and without ATRA treatment at T = 24 hr. Blot corresponds to Fig 8B, fourth row, second column.

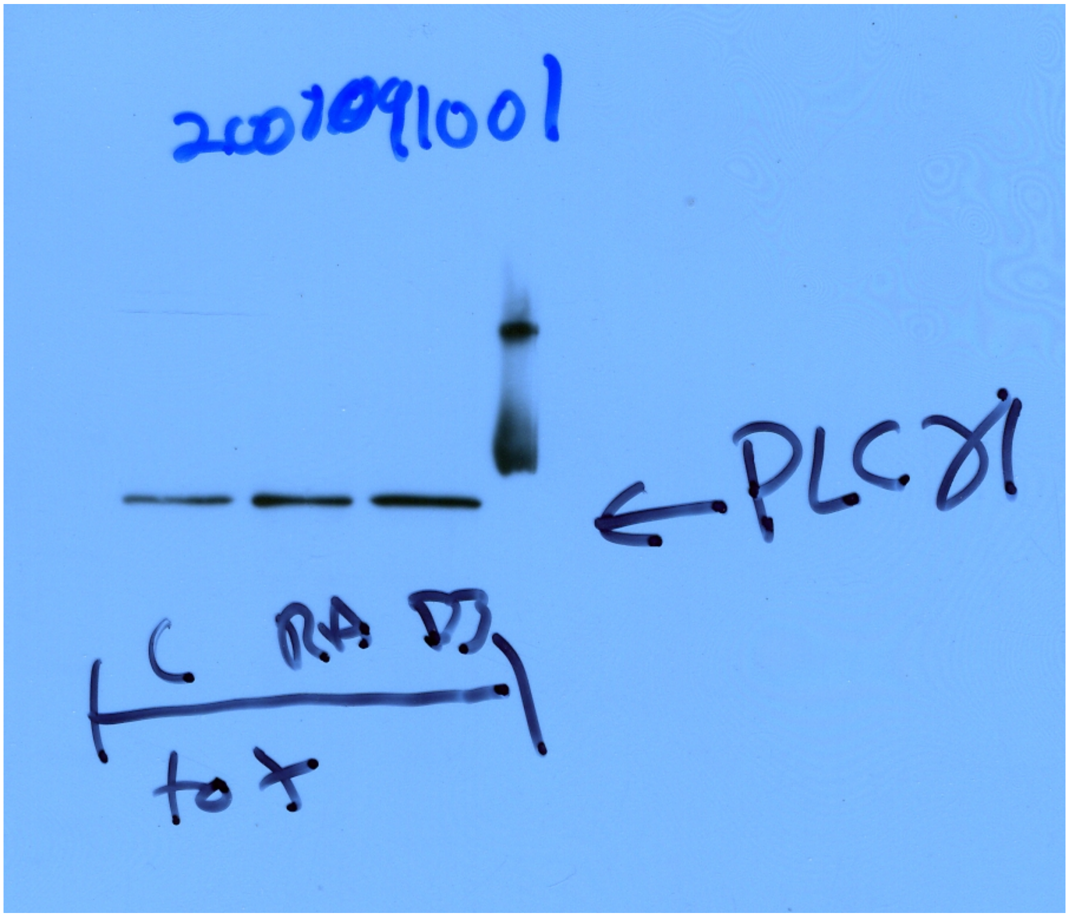

Fig. S24: Western blot (total lysate) for PLCγ with and without ATRA treatment at T = 24 hr. Blot corresponds to Fig 8B, sixth row, second column.

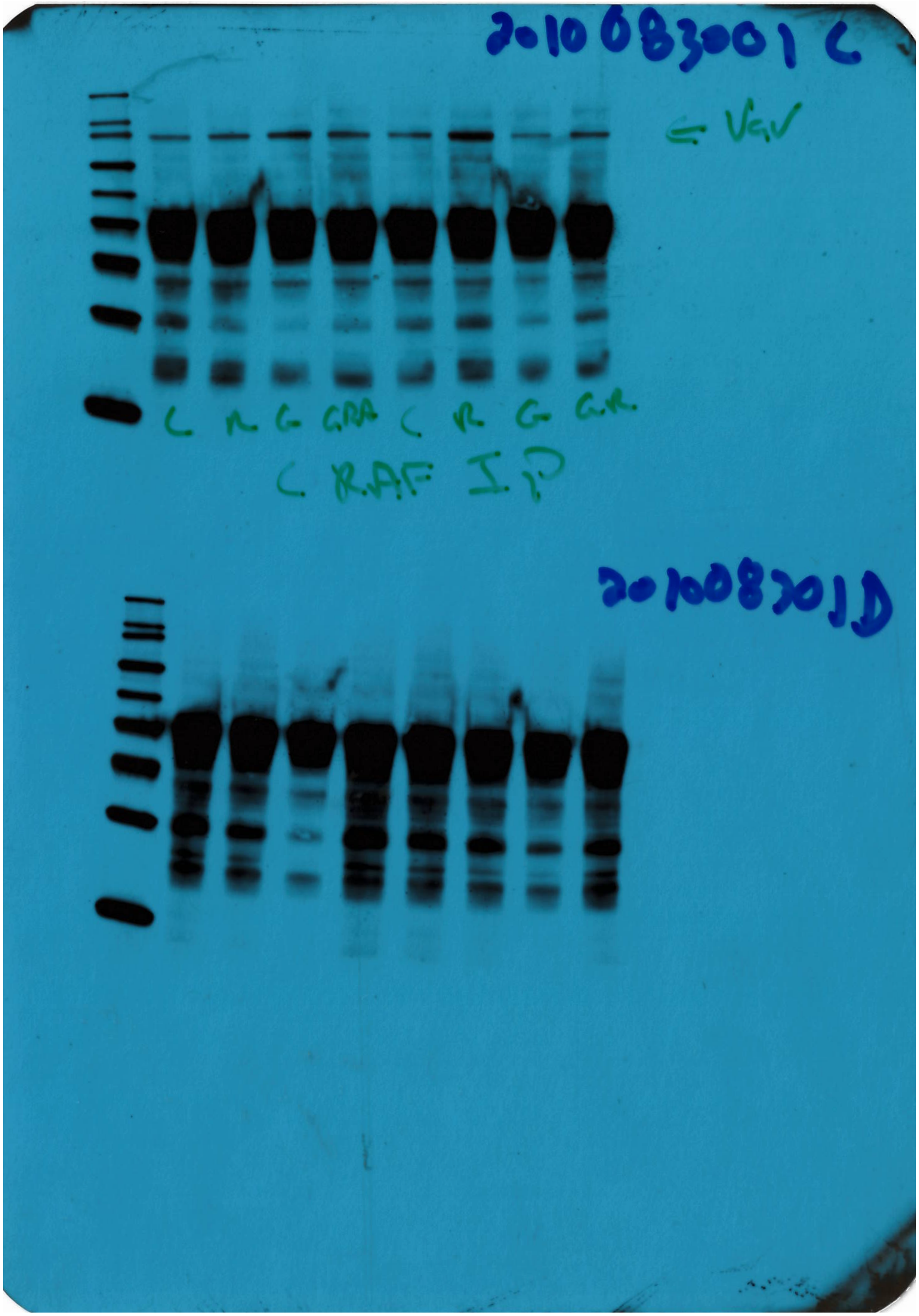

Fig. S25: Vav1 immunoprecipitation with cRaf with and without ATRA treatment and the Raf inhibitor GW5074 at T = 24 hr. Blot (right hand replicate) corresponds to Fig 8C, first row

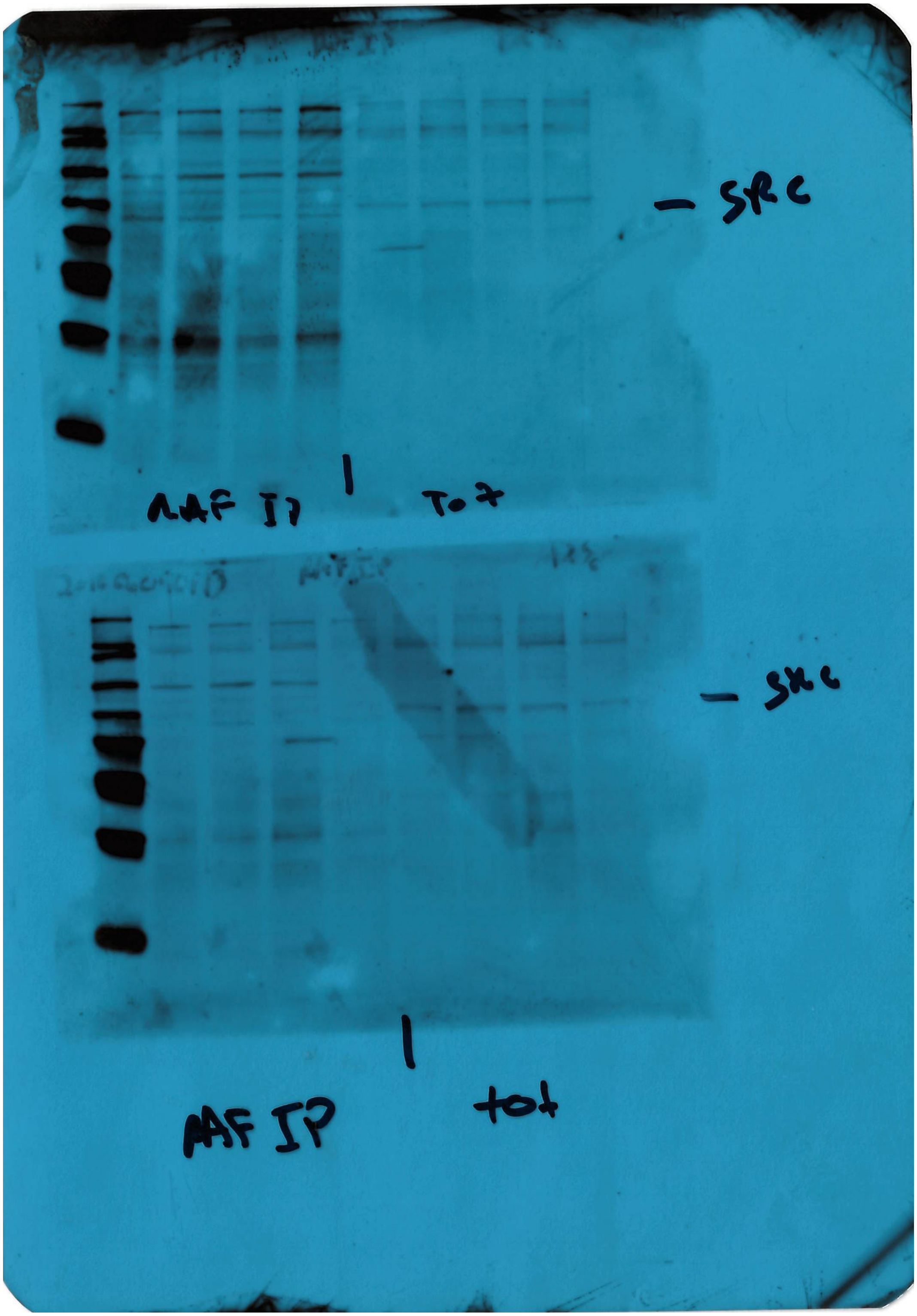

Fig. S26: Src immunoprecipitation with cRaf, and Src (total lysate) with and without ATRA treatment and the Raf inhibitor GW5074 at T = 24 hr (bottom replicate). Blot corresponds to Fig 8C, second row. Lanes: control (no ATRA or GW); ATRA alone; GW5074 alone; ATRA and GW5074.

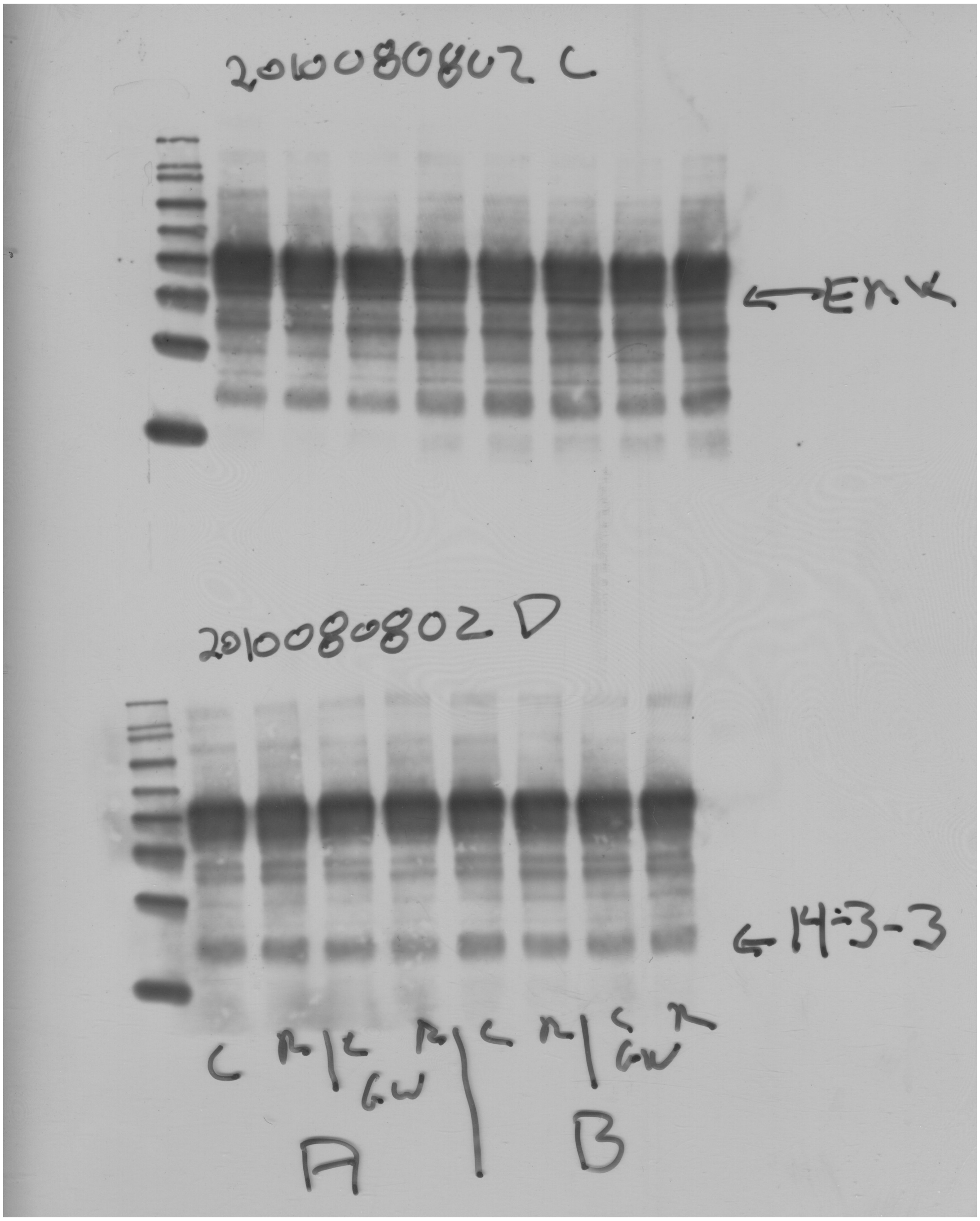

Fig. S27: 14-3-3 immunoprecipitation with cRaf with and without ATRA treatment and the Raf inhibitor GW5074 at T = 24 hr. Blot corresponds to Fig 8C, third row

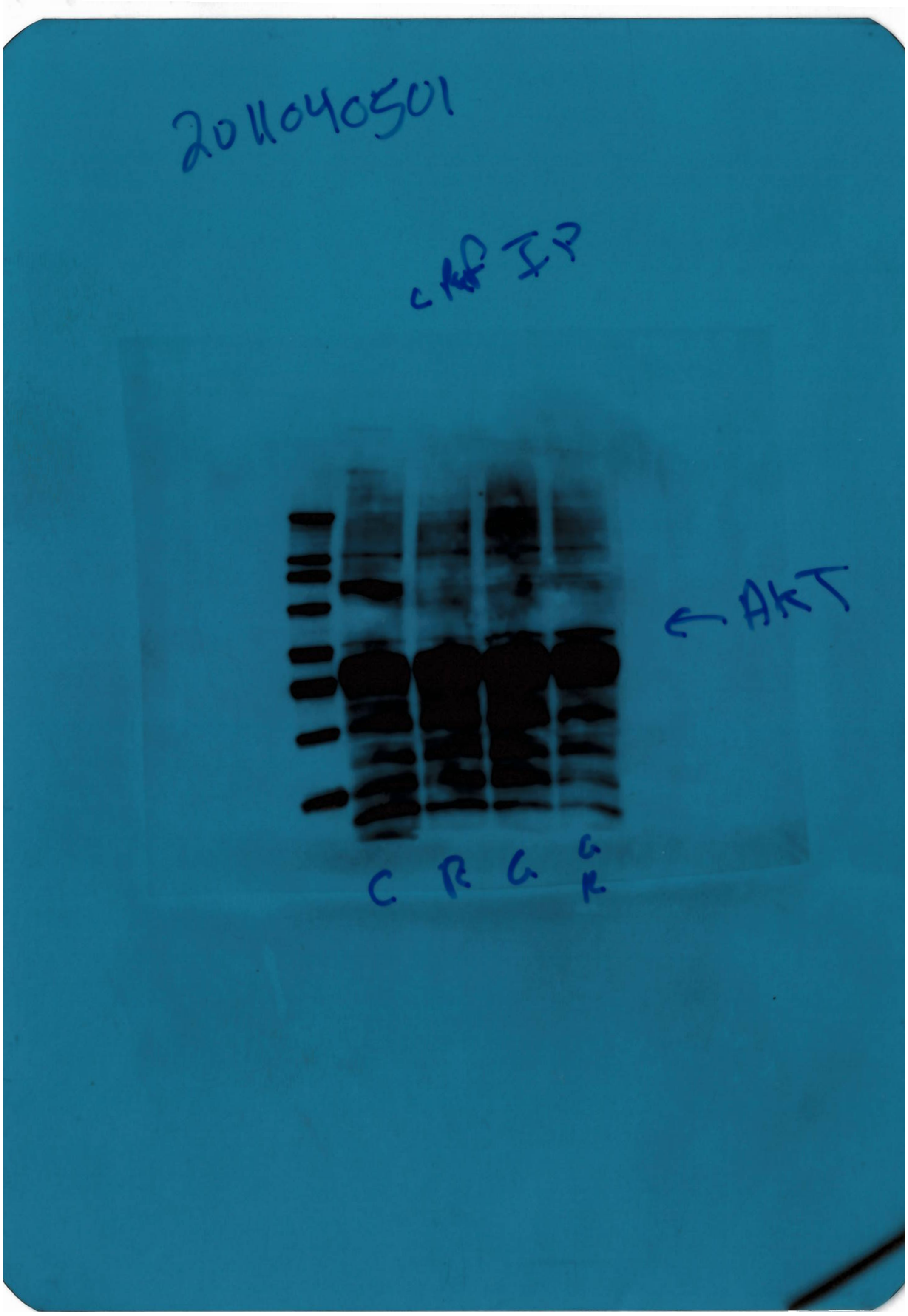

Fig. S28: Akt immunoprecipitation with cRaf with and without ATRA treatment and the Raf inhibitor GW5074 at T = 24 hr. Blot corresponds to Fig 8C, fourth row

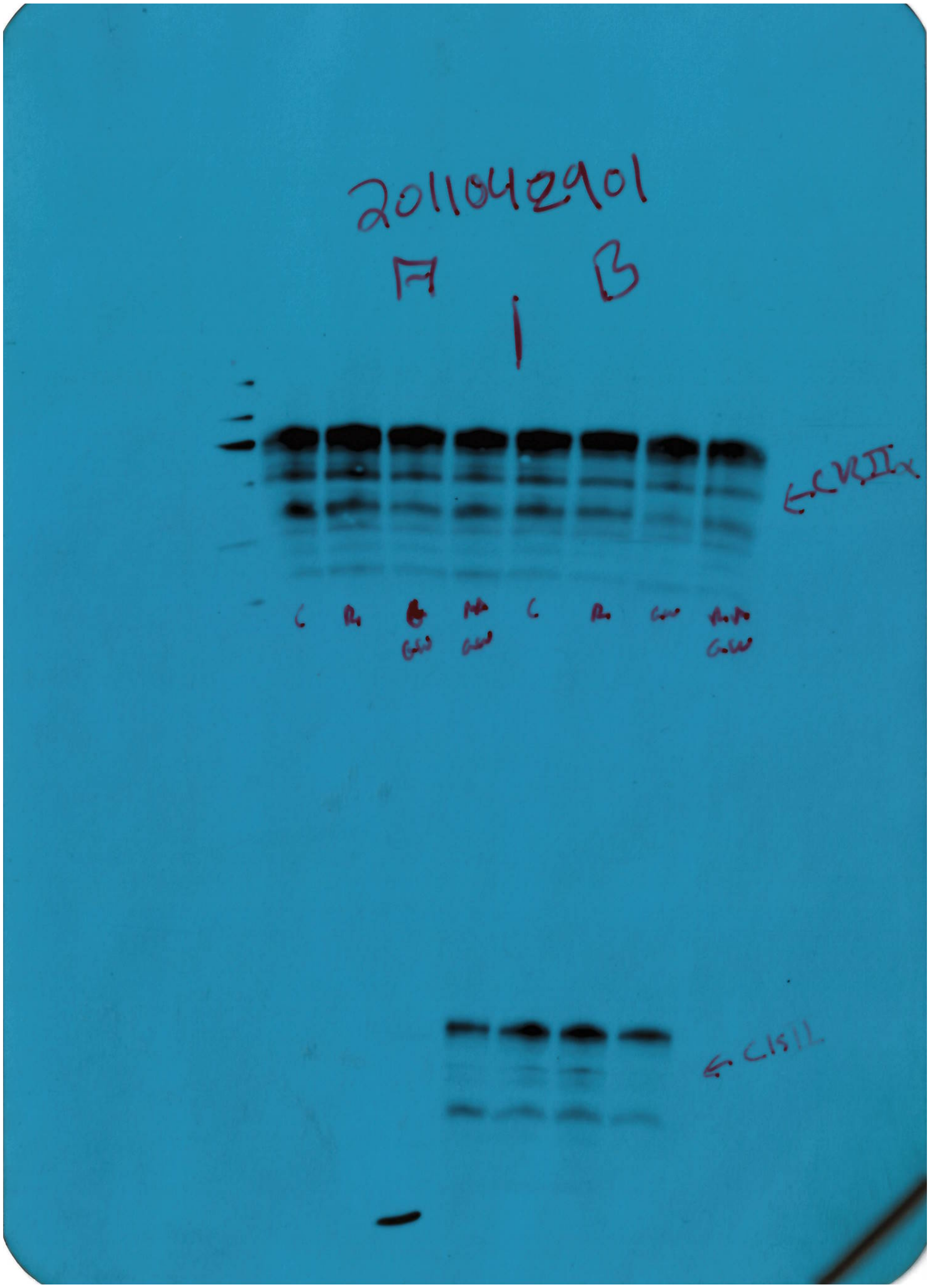

Fig. S29: CK2 immunoprecipitation with cRaf with and without ATRA treatment and the Raf inhibitor GW5074 at T = 24 hr. Blot (right-hand replicate) corresponds to Fig 8C, fifth row

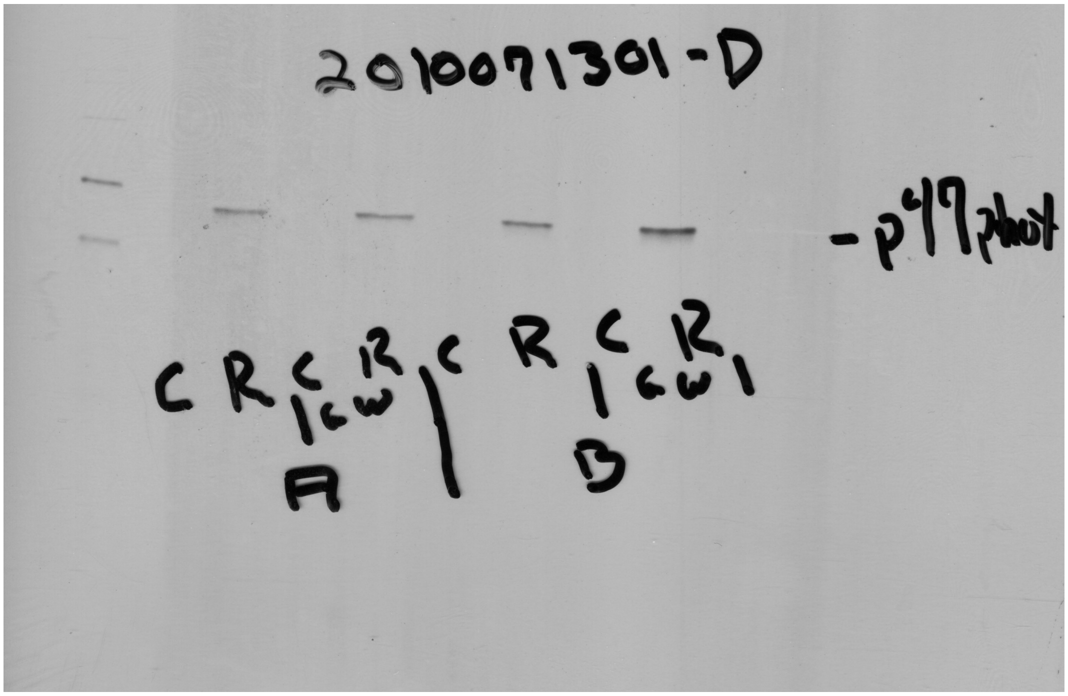

Fig. S30: Western blot for P47Phox (total lysate) with and without ATRA treatment and the Raf inhibitor GW5074 at T = 48 hr. Blot (left-hand replicate) corresponds to Fig 8F.
